# Microarray Analysis of the Effect of *Streptococcus equi* subsp. *zooepidemicus* M-Like Protein in Infecting Porcine Pulmonary Alveolar Macrophage

**DOI:** 10.1371/journal.pone.0036452

**Published:** 2012-05-02

**Authors:** Zhe Ma, Hui Zhang, Li Yi, Hongjie Fan, Chengping Lu

**Affiliations:** College of Veterinary Medicine, Nanjing Agriculture University, Nanjing, China; University of Kansas Medical Center, United States of America

## Abstract

*Streptococcus equi* subsp. *zooepidemicus* (*S.* z*ooepidemicus*), which belongs to Lancefield group C streptococci, is an important pathogen of domesticated species, causing septicemia, meningitis and mammitis. M-like protein (SzP) is an important virulence factor of *S. zooepidemicus* and contributes to bacterial infection and antiphagocytosis. To increase our knowledge of the mechanism of SzP in infection, we profiled the response of porcine pulmonary alveolar macrophage (PAM) to infection with *S. zooepidemicus* ATCC35246 wild strain (WD) and SzP-knockout strain (KO) using the Roche NimbleGen Porcine Genome Expression Array. We found SzP contributed to differential expression of 446 genes, with upregulation of 134 genes and downregulation of 312 genes. Gene Ontology category and KEGG pathway were analyzed for relationships among differentially expressed genes. These genes were represented in a variety of functional categories, including genes involved in immune response, regulation of chemokine production, signal transduction and regulation of apoptosis. The reliability of the data obtained from the microarray was verified by performing quantitative real-time PCR on 12 representative genes. The data will contribute to understanding of SzP mediated mechanisms of *S.* z*ooepidemicus* pathogenesis.

## Introduction

*Streptococcus equi* subsp. *zooepidemicus* (*S.* z*ooepidemicus*), a member of the Lancifield's group C, is primarily an opportunistic pathogen infecting a wide variety of animal species, including important domestic species such as horses, cows, pigs, sheep, and dogs; as such, it is a pathogen of veterinary concern. It is a well-known cause of mastitis in cows and mares, and is the most frequently isolated opportunistic pathogen of horses [1]. In China, it is an important swine pathogen, causing septicemia, meningitis, endocarditis and arthritis, resulting in significant economic losses worldwide [2], [3]. Humans may contract *S.* z*ooepidemicus* after consuming contaminated food or from close contact with infected animals [4]–[6]. In 1975, Sichuan province experienced a *S.* z*ooepidemicus* pandemic, resulting in the death of 300,000 pigs and great economic losses. It is the second most important pathogen for streptococcal diseases in swine [7], [8], and to date, it remains a great threat to the chinese pig industry.

Prior to the establishment of infection in a non-immune host, pathogenic microorganisms must evade the innate immune system [9], thus, many pathogens have unique surface structures which interfere with phagocytosis by neutrophils [10]. M protein is an important virulence factor of group A streptococci; this fibrillar, surface-exposed protein interferes with alternate complement pathway mediated opsonization of the organism [11], [12]. Previous studies have demonstrated that *S. zooepidemicus* carry antigens with antiphagocytic properties similar to the M proteins from Lancefield group A and G streptococci, thus it was named M-like protein (SzP) [13]. SzP is a cell surface-anchored protein that confers resistance to phagocytosis [14], with SzP-knockout strains having a1000-fold decrease in 50% lethal dose (LD_50_) value compared with the wild type strain [15]. However, the molecular mechanisms by which SzP protects *S. zooepidemicus* from phagocytosis are poorly understood.

Host-pathogen interactions during *S. zooepidemicus* infection are complex; in recent years, microarrays have been widely used to study these complex molecular mechanisms underlying the host response to pathogenic microorganisms in macrophages. The aim of this study was to profile differences in gene expression for the porcine pulmonary alveolar macrophage (PAM) infected with *S. zooepidemicus* ATCC35246 wild strain (WD) or SzP-knockout strain (KO) to elucidate mechanisms of SzP during *S. zooepidemicus* infection.

## Results

### Microarray analysis

To investigate the molecular mechanisms of SzP during *S. zooepidemicus* infection, the differential gene expression profile of PAM cells was determined after infection with *S. zooepidemicus* WD or KO. Gene upregulation was defined as a fold change in relative transcription levels Log FC≥1 and FDR≤0.05; similarly, downregulation was defined as an Log FC≤−1 and FDR≤0.05. Genes with relative transcription levels of −1≤Log FC≤1 were considered to show no notable change. In this study, 446 transcripts showed a level of expression that differed significantly from that of the control group infected with active *S. zooepidemicus* KO (Table S1).

### Gene Ontology category

**Figure 1:**
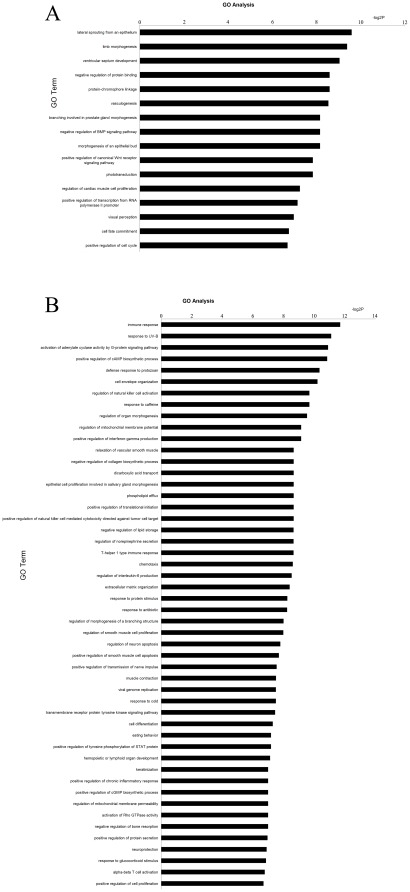
GO category based on biological process for differentially expressed genes. (A) The significant GO category for upregulated genes. (B) The significant GO category for downregulated genes. P-value<0.05 and FDR<0.05 were used as a threshold to select significant GO categories. LgP is the logarithm of P-value.

The 446 differentially expressed genes were classified into different functional categories according to Gene Ontology (GO) project for biological process; among these, 134 genes were upregulated and 312 genes were downregulated. The main GO categories for upregulated genes were response to negative regulation of protein binding, negative regulation of BMP signaling pathway, positive regulation of canonical Wnt receptor signaling pathway, regulation of cardiac muscle cell proliferation, positive regulation of transcription from RNA polymerase II promoter, positive regulation of cell cycle (Fig. 1A). The primary GO categories for downregulated genes were immune response, activation of adenylate cyclase activity by G-protein signaling pathway, positive regulation of cAMP biosynthetic process, defense response to protozoan, regulation of natural killer cell activation, positive regulation of interferon-gamma production, positive regulation of natural killer cell mediated cytotoxicity directed against tumor cell target, T-helper 1 type immune response, chemotaxis, regulation of interleukin-6 production, response to protein stimulus, transmembrane receptor protein tyrosine kinase signaling pathway, positive regulation of tyrosine phosphorylation of STAT protein, positive regulation of chronic inflammatory response, regulation of mitochondrial membrane permeability, activation of Rho GTPase activity, positive regulation of protein secretion, alpha-beta T cell activation (Fig. 1B). The differentially expressed genes involved in significant GO categories are summarized in Table S2. The wide diversity in these categories suggests that SzP of *S. zooepidemicus* has a significant impact on the physiology and function of host cells during infection.

### Pathway analysis

**Figure 2:**
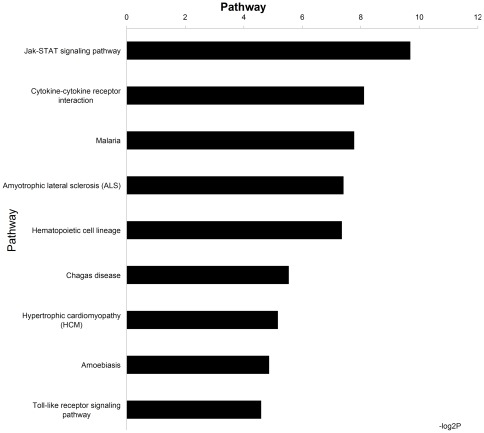
KEGG pathway analysis for differentially expressed genes. The significant pathway for downregulated genes. P-value<0.05 and FDR<0.05 were used as a threshold to select significant KEGG pathways. LgP is the logarithm of P-value to the base 10.

The KEGG pathway analysis for DE genes showed that only some downregulated genes could connect to each other in a pathway specific manner, whereas none of the upregulated genes could be connected to form pathways. These downregulated genes were involved in Jak-STAT signaling pathway, Cytokine-cytokine receptor interaction, Malaria, Amyotrophic lateral sclerosis (ALS), Hematopoietic cell lineage, Chagas disease, Hypertrophic cardiomyopathy (HCM), Amoebiasis, Toll-like receptor signaling pathway (Fig. 2). The downregulated genes involved in significant pathways are summarized in Table S3.

### STRING analysis of the relationships between DE genes

**Figure 3:**
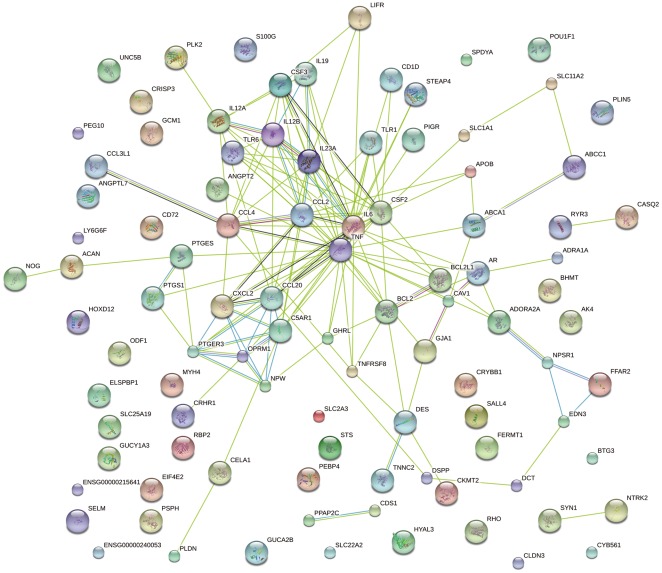
STRING analysis of the relationship between DE genes. The DE genes between PAM infected with S. zooepidemicus WD and KO were analyzed using the STRING database (http://string90.embl.de). The network nodes represent the proteins encoded by the DE genes. Seven different colored lines link a number of nodes and represent seven types of evidence used in predicting associations. A red line indicates the presence of fusion evidence; a green line represents neighborhood evidence; a blue line represents coocurrence evidence; a purple line represents experimental evidence; a yellow line represents textmining evidence; a light blue line represents database evidence and a black line represents coexpression evidence. (For interpretation of the references to color in this figure legend, the reader is referred to the web version of the article.)

DE genes between PAM infected with *S. zooepidemicus* WD and KO were analyzed using STRING (http://string90.embl.de), a database of known and predicted protein interactions. Fig. 3 summarizes the network of predicted associations for DE gene encoded proteins. The results indicate that genes TNF, TLR1, TLR6, IL23A, IL6, IL12A, CSF2, CSF3, BCL2L1 and IL12B are associated according to experimental evidence, with involvement in many signaling pathways and other immune responses; the genes PTGER3, OPRM1, NPW, C5AR1, CCL20 and CXCL2 encoding genes are also related. TNF was the key gene of this protein interaction net, linking to CCL4, CAV1, TLR1, BCL2, etc, and these genes linked to many downstream genes, all of these genes are inter-related, forming a large network. However, many genes are not linked to other genes, indicating that there functions are unrelated or unknown.

### Confirmation of microarray results by quantitative real-time PCR

Microarray experiments yield a large amount of data, therefore it is important to validate differential expression by independent methods. In order to verify the data obtained by microarray analysis, quantitative real-time PCR was performed on 12 differentially expressed genes (5 upregulated and 7 downregulated). Total RNA was purified from infected PAM (same preparation method as the RNA for the microarrays). The results for the 12 selected genes showed that the direction of expression changes were consistent with those found by microarray analysis. The fold-changes determined by microarray and real-time RT-PCR, however, were different. This may be due to technical differences in the methods of analysis and normalization. As shown in Table 1, the results confirmed the data from the microarray.

**Table 1:** Real-time quantitative RT-PCR.

	GenBank ID	Gene	Microarray LogFC	Real-time RT-PCR LogFC
1	NM_001160075.1	CLDN3	2.47	1.41
2	XM_001925690.1	RYR3	1.89	5.03
3	NM_214255.1	ODFP	1.7	2.51
4	NM_214438.2	CAV1	1.21	2.13
5	DQ915497.1	CRYBB1	1.02	4.12
6	DQ779285.1	PEG10	−1.05	−3.56
7	AB271960.1	BCL2	−1.11	−1.25
8	EW025078	GHRL	−1.43	−1.23
9	NM_001164652.1	ACAN	−1.56	−2.14
10	NM_213993.1	IL12A	−2.29	−5.36
11	DN112929	CXCL2	−2.54	−3.25
12	NM_214399.1	IL6	−3.1	−2.86

## Discussion

*S. zooepidemicus* has developed a multitude of strategies to evade host defenses by interfering with many aspects of the immune system. *S. zooepidemicus SzP* is a 40.1-kD cell surface-anchored protein that elicits serum opsonic and protective responses in mice and horses [14]. Previous studies have indicated that SzP and M proteins in the Lancefield groups A and G streptococci share similar antiphagocytic characteristics [16]. In this study, we found 446 transcripts with significant differential expression in response to PAM infection with *S. zooepidemicus* WD or KO, of which, 134 genes were upregulated and 312 genes were downregulated. Gene Ontology category and KEGG pathway results indicated these differentially expressed genes belong to a variety of functional categories and signal pathways.

These downregulated genes were associated with immune response, regulation of natural killer cell activation, T-helper 1 type immune response, regulation of interleukin-6 production, positive regulation of protein secretion, and alpha-beta T cell activation, which may have helped to clear the pathogen when induced; downregulation of these genes and reduction of their associated functions likely aided in the infection of *S. zooepidemicus*. Recent studies have revealed the important regulatory roles played by chemokines and their receptors in inflammatory reactions. We observed that several chemokines were differentially expressed in *S. zooepidemicus* infected PAM, comparing WD and KO treatments. Chemokine (C-C motif) ligand 2 (CCL2), also known as Monocyte chemoattractant protein 1 (MCP-1), influences both innate immunity through monocytes, and adaptive immunity by regulating T helper cell polarization [17]. Induction of monocyte recruitment has been found in a number of different pathologies, including infection. Mice lacking MCP-1 have an increased susceptibility to an interstitial polymicrobial infection [18]. The key protein regulating the immune and inflammatory response in most streptococci infected tissues is considered to be TNF-α (Tumor Necrosis Factor-alpha) [19]–[22]. A recent study has revealed that Mycobacterium tuberculosis potentially increases its virulence by inhibiting TNF biosynthesis in human macrophages, which results in the subversion of host immunity [23]. In addition, TNF-α may increase macrophage phagocytosis as a host defense against bacteria [24]. Colony stimulating factor 3 (CSF3/G-CSF) is an important survival and proliferation factor for neutrophils and macrophages. Adult macrophages treated with recombinant human CSF3 showed significant increases in killing of group B streptococcus [25]. In our study, MCP-1, TNF-α and CSF3 of WD infected PAM were all downregulated, when compared with KO infection, which is likely to increase *S. zooepidemicus* pathogenesis. Low levels of TLR rendered PAM refractory to activation by bacterial components and reductions in TNF-α helped bacteria avoid phagocytosis. There was a report indicating that thioredoxin-1 (TRX) catalytically interacted with tumor necrosis factor receptor superfamily member 8 (TNFRSF8/CD30) as a single principal target protein [26]. Our previous research showed that SzP may interact with TRX [27]; the microarray data showed that SzP downregulated CD30, thus we presumed there should be some relationship among SzP, TRX and CD30, which warrants further study.

Pathway analysis results showed that only a subset of downregulated genes were related to signaling pathways. We found that IL12A, which is related to the Jak-STAT signaling pathway, was downregulated. A report indicated that silencing of IL12A in dendritic cells (DCs) decreased IL12-induction of T cell responses by blocking tyrosine phosphorylation of JAK2, TYK2, STAT3, and STAT4 proteins; downregulation of IL12A modulated immune responses by blocking IL12 signaling through JAK-STAT signaling pathway in T cells [28]. The JAK-STAT signaling pathway is involved in immune function and cell growth, and is the principal signaling mechanism for many cytokines and growth factors in mammals [29]. Cytokine signaling through the JAK/STAT pathway regulates multiple cellular responses, thus significant attention has been focused on the role of cytokines during inflammation and immunity.

Innate immunity is the first line of defense for host protection against invading pathogens. Our results indicated that the Toll-like receptor signaling pathway was downregulated in WD *S. zooepidemicus* infection, compared with KO. Toll-like receptors (TLRs) are receptors that play a key role in the innate immune response, whose activation results in a signaling cascade, ultimately releasing pro-inflammatory cytokines [30]. TLR is the receptor for lipoteichoic acid (LTA), a cell wall component from diverse Gram-positive bacterial species [31]. Previous studies have shown that TLR2/TLR6 and TLR9 recognize *S. suis*, which results in the release of pro-inflammatory mediators. Interactions of lipoproteins with TLR6 likely increase activity of NF-kB [32]. In our study, TLR6 was downregulated in the presence of SzP from *S. zooepidemicus* infection, which likely increases pathogenesis of *S. zooepidemicus* by inhibiting the host immune response, in addition to escaping from phagocytosis.

Some DE genes, such as TNFRSF11B, IL23RA, IL23A/B and CSF2/3, are involved in the Cytokine-cytokine receptor interaction. Cytokines are soluble extracellular proteins or glycoproteins which regulate critical aspects of cell regulation and mobilization for innate and adaptive inflammatory host defenses [33]. BCL2 regulates cell apoptosis and necrosis by controlling mitochondrial membrane permeability and is involved in the apoptosis signaling pathway [34]. Prevoius work showed *S. suis 2* infection up regulated BCL2 gene expression in human cells [35], but in our results, this gene was down regulated in the presence of SzP; this mechanism needs more experiments to explain. In addition, our results suggested that SzP from *S. zooepidemicus* infection affects multiple signal transduction pathways; further studies are needed to fully describe the pathways involved in the host cell response to SzP in *S. zooepidemicus* infection.

In summary, we performed global differential expression analysis between *S. zooepidemicus* WD and KO infected PAM, with the aim of understanding the mechanisms by which SzP help *S. zooepidemicus* to evade the host immune system. Many of the genes identified during this analysis were of unknown function or were even unannotated, thus, our results provide gene expression profiles for use in future research into the molecular mechanisms involved in *S. zooepidemicus* pathogenesis and in swine diagnosis, prevention and treatment of *S. zooepidemicus* infection.

## Methods and Materials

### Ethics

Porcine PAM was obtained with consent from one healthy pig under ethical approval granted by the Nanjing Agricultural University Veterinary College. The protocol was approved by the Science and Technology Agency of Jiangsu Province. All efforts were made to minimize animal's suffering. The approval ID is SYXK (SU) 2010-0005, granted by the Nanjing Agricultural Veterinary College Experimental Animal ethics committee.

### Bacterial strains and growth conditions

*S. zooepidemicus* strain ATCC35246 wild strain (WD) was isolated from diseased pigs in the Sichuan province of China. *S. zooepidemicus* M-like protein (SzP) knockout strain (KO) was constructed by our lab [15]. Bacteria were grown on blood agar plates for 18 h at 37°C, and inoculated into THB medium (Bacton™, USA) for 6 h at 37°C. Bacteria were pelleted by centrifugation at 13,000 rpm for 10 min, washed twice with phosphate-buffered saline (PBS, pH 7.4), and then diluted to approximately 1×10^9^ colony-forming units (CFU)/mL.

### Porcine PAM isolation and culture conditions

Porcine alveolar macrophages obtained from 4–8 week old pigs were prepared as previously described [36], [37]. DMEM medium and fetal bovine serum (FBS) were obtained from GIBCO (Invitrogen). The isolated cells were grown and maintained in DMEM medium containing 10% (v/v) FBS and at 37°C in a 5% CO_2_ atmosphere.

### Exposure of cells to bacteria

For the stimulation assays, 6 h-cultures of PAM cells were washed twice with PBS and resuspended in DMEM maintenance medium. *S. zooepidemicus* WD and KO were diluted appropriately in maintenance medium respectively, and added to PAM cells at a multiplicity of infection (MOI) of 10∶1 (bacteria/cells). Uninfected control cells were incubated with DMEM maintenance media only (mock infected). After a 3 h incubation period, cells were collected by centrifugation at 1000 g for 10 min and the supernatant was then discarded.

### RNA preparation

After exposure to bacteria, cells were centrifuged in RNase-free tubes treated with Trizol Reagent (Invitrogen, Carlsbad, CA, USA) and stored at −80°C prior to RNA extraction. Total RNA extraction from cells was performed according to the manufacturer's standard instructions (Invitrogen) and then the RNA was prepared and purified using the NucleoSpin® RNA clean up kit (MACHEREY-NAGEL, Germany). RNA concentration was assessed by Nanodrop 2000 spectrophotometry (Thermo). RNA quality was determined by formaldehyde denaturation electrophoresis and only those samples showing no degradation (ratios approaching 2∶1 for the 28S and 18S bands) were used to generate labeled targets.

### Microarray hybridization and data analysis

The RNA samples were sent to CapitalBio Beijing, China, for microarray hybridization. Each RNA sample from different PAM treatments was hybridized to one Roche NimbleGen Porcine Genome Expression Array (Roche). Briefly, double-stranded cDNA was synthesized from 6 mg of total RNA using a T7-oligo (dT) primer. The cDNA was further purified and converted into cRNA using an in vitro transcription reaction. 5 µg cRNA was reverse transcribed to cDNA, fragmented, and then labeled with Cy3-dCTP (GE Healthcare) using Klenow. These labeled cDNA fragments were hybridized to NimbleGen Porcine Genome Expression Arrays for 16 h at 42°C using the Roche NimbleGen Hybridization System 12. Afterwards, the GeneChips were washed, stained, and then scanned with a Roche-NimbleGen MS200. The Roche NimbleGen Porcine Genome Expression Array contains over 135,000 probe sets, representing 45,023 transcripts and variants, including 7858 well-characterized porcine genes.

The hybridization data were analyzed using GeneChip Operating Software (GCOS 1.4). The scanned images were first assessed by visual inspection, then analyzed to generate raw data files saved as CEL files using the default setting of GCOS 1.4. RMA (Robust Multichip Analysis) was used to normalize the different arrays. To find differentially expressed genes, we used Linear models and empirical Bayes methods to analyze the data [38]. The method is similar to a standard t-test for each probe except that the SES is moderated across genes to get more stable results, preventing a gene with a very small change from being judged as differentially expressed due to an accidentally small residual SD. The resulting P values were adjusted using the BH FDR algorithm [39]. Differential expression of genes was considered significant if both the FDR values<0.05 (controlling the expected FDR to no more than 5%) and the fold change ≥|1.5|.

### STRING analysis, GO category and pathway analysis

Differentially expressed (DE) genes in porcine PAM infected with WD or KO were analyzed using STRING (http://string-db.org/), a database of known and predicted protein interactions.

We downloaded the GO annotations for the Microarray genes from NCBI (http://www.ncbi.nlm.nih.gov/), UniProt (http://www.uniprot.org/) and the Gene Ontology (http://www.geneontology.org/). The “elim Fisher" algorithm described by Adrian Alexa et.al was used for the GO enrichment test because it can iteratively remove any genes mapped to significant GO terms from more general (higher level) GO terms, preventing the general GO terms from masking the significant terms [40]. Gene ontology categories with a p-value<0.05 were reported.

Pathway analysis was used to determine significant pathways for differentially expressed genes using microarray gene pathway annotations downloaded from KEGG (http://www.genome.jp/kegg/). A Fisher exact test was used to find significant enrichment for pathways and the resulting P values were adjusted using the BH FDR algorithm [39]; pathway categories with a FDR<0.05 were reported. Enrichment provides a measure of the significance of the function: as the enrichment increases, the corresponding function is more specific, which helps us to find pathways of greater significance in the experiment. The enrichment was given by: Reenrichment = (n_g_/n_a_)/(N_g_/N_a_) where n_g_ is the number of differentially expressed genes within the particular pathway, n_a_ is the total number of genes within the same pathway, N_g_ is the number of differential genes which have at least one pathway annotation, and N_a_ is the number of genes which have at least one pathway annotation in the entire microarray.

### Real-time PCR

Real-time quantitative PCR was used to validate selected data from the microarray experiments and to follow the expression of a subset of genes over time. Total RNA was reverse transcribed using the PrimeScript™ reagent Kit (Takara) according to the manufacturer's instructions, with GAPDH serving as an endogenous control. The genes used for the RT-PCR assays are listed in Table 1. The cDNA samples were used for quantitative real-time PCR analysis, which was performed using a 7300 Real-Time PCR System (ABI) with SYBR Premix Ex Taq™ (Takara) according to the manufacturer's instructions. Amplification conditions were 95°C for 10 s, followed by 40 cycles of 95°C for 5 s and 60°C for 31 s. Each sample was run in triplicate alongside the endogenous control to normalize reactions. After completion of the PCR amplification, the relative fold change after stimulation was calculated based on the 2^−ΔΔCT^ method [41].

## Supporting Information

Table S1 Differential gene expression profile of PAM cells after infection with *S. zooepidemicus* WD or KO.
(XLS)

Table S2 The differentially expressed genes involved in significant GO categories.
(XLSX)

Table S3 The downregulated genes involved in significant pathways.
(XLS)
