# Correction: Microarray Analysis of the Effect of *Streptococcus equi* subsp. *zooepidemicus* M-Like Protein in Infecting Porcine Pulmonary Alveolar Macrophage

**DOI:** 10.1371/annotation/33056b27-c9ab-46ef-8168-08a8f0f907b2

**Published:** 2012-05-18

**Authors:** Zhe Ma, Hui Zhang, Li Yi, Hongjie Fan, Chengping Lu

Funding should read "This study was supported by Program from the National Basic Research Program (973) of China (2012CB518804), the National Natural Science Foundation of China (31172319), the key Technology R?D Program of Jiangsu (BE2009388), the Fundamental Research Funds for the Central Universities (KYT 201003), and the Project Funded by the Priority Academic Program Development of Jiangsu Higher Education Institutions. The funders had no role in study design, data collection and analysis, decision to publish, or preparation of the manuscript." 

